# Development of a Cannabinoid-Based Photoaffinity Probe to Determine the Δ^8/9^-Tetrahydrocannabinol Protein Interaction Landscape in Neuroblastoma Cells

**DOI:** 10.1089/can.2018.0003

**Published:** 2018-07-01

**Authors:** Marjolein Soethoudt, Georgios Alachouzos, Eva J. van Rooden, María Dolores Moya-Garzón, Richard J.B.H.N. van den Berg, Laura H. Heitman, Mario van der Stelt

**Affiliations:** ^1^ Department of Molecular Physiology, Leiden Institute of Chemistry, Leiden University, Leiden, The Netherlands.; ^2^ Division of Drug Discovery and Safety, Leiden Academic Centre for Drug Research, Leiden University, Leiden, The Netherlands.; ^3^ Bio-Organic Synthesis, Leiden Institute of Chemistry, Leiden University, Leiden, The Netherlands.

**Keywords:** photoaffinity labeling, chemical proteomics, tetrahydrocannabinol, cannabinoid receptors, protein targets

## Abstract

**Introduction:** Δ^9^-Tetrahydrocannabinol (THC), the principle psychoactive ingredient in *Cannabis*, is widely used for its therapeutic effects in a large variety of diseases, but it also has numerous neurological side effects. The cannabinoid receptors (CBRs) are responsible to a large extent for these, but not all biological responses are mediated via the CBRs.

**Objectives:** The identification of additional target proteins of THC to enable a better understanding of the (adverse) physiological effects of THC.

**Methods:** In this study, a chemical proteomics approach using a two-step photoaffinity probe is applied to identify potential proteins that may interact with THC.

**Results:** Photoaffinity probe **1**, containing a diazirine as a photocrosslinker, and a terminal alkyne as a ligation handle, was synthesized in 14 steps. It demonstrated high affinity for both CBRs. Subsequently, two-step photoaffinity labeling in neuroblastoma cells led to identification of four potential novel protein targets of THC. The identification of these putative protein hits is a first step towards a better understanding of the protein interaction profile of THC, which could ultimately lead to the development of novel therapeutics based on THC.

## Introduction

Preparations of the plant *Cannabis sativa* have been used throughout history in various cultures as medicinal concoctions or therapeutics, as well as for recreational or religious purposes.^1^ In 1930, the isolation of cannabinol and cannabidiol as the first active substituents was achieved,^2^ which was followed by the discovery of Δ^9^-tetrahydrocannabinol (THC) in 1964.^3^ THC is the psychoactive constituent of marijuana and exists in two isomers: namely Δ^9^-THC and Δ^8^-THC, of which the latter is the most thermodynamically stable isomer.^4^

THC treatment has been associated with therapeutic effects, such as analgesia, relaxation and fatigue, appetite stimulation,^5^ antiemesis,^6^ and reduction of nausea.^5^ THC is used by patients suffering from multiple sclerosis (MS),^7^ cancer, or AIDS.^8^ In addition, preclinical data of THC indicate beneficial effects in several animal models of Alzheimer's,^9^ Parkinson's,^10^ and Huntington's disease.^11^ However, THC is also associated with many undesirable side effects, including induction of psychoactivity, anxiety, memory loss, cardiac arrhythmias, and addiction.^12^

Both Δ^9^-THC and Δ^8^-THC have similar affinity to the cannabinoid receptor type 1 (CB_1_R) and type 2 (CB_2_R).^13,14^ The CB_1_R is the most abundant G protein-coupled receptor (GPCR) in the mammalian brain,^15^ whereas the CB_2_R is predominantly present in peripheral tissues and cells of the immune system.^16^ Most of the physiological effects of THC are mediated via the CB_1_R and CB_2_R as demonstrated by the use of specific CB receptor antagonists or genetically modified mice that lack the CB receptors.^17–20^

It is, however, hypothesized that THC may have other non-CB receptor targets. A study, using CB_1_R and CB_2_R knockout mice, showed similar analgesia upon THC administration compared with the equivalent wild-type mice in the tail-flick test.^21^ This effect was not observed in the hotplate test, which requires spinal processing of nociceptive information. These observations suggest the existence of another protein target in the brain. Previously, orphan GPCRs GPR55 and GPR18 and peroxisome proliferator-activated receptor gamma were identified to bind to THC, but it is unclear whether these targets are responsible for some of the physiological effects of THC.^22–24^ Therefore, a more complete view of the protein interaction of THC in neuronal cells is desirable.

Photoaffinity-based protein profiling (pA*f*BPP) has been previously used to map the protein interaction landscape of small molecules.^25,26^ Photoaffinity probes use a light-responsive element to covalently crosslink the compound with its target protein upon irradiation. To circumvent the problems associated with large reporter groups, photoaffinity probes with a bioorthogonal ligation handle (e.g., alkyne), to introduce a fluorescent or affinity tag (e.g., biotin) after crosslinking to a protein, have emerged as powerful tools to visualize small molecule-protein interactions in living systems.^27^ Previously, we applied two-step pA*f*BPP to capture and visualize the CB_2_R on human cells.^28^ Here, it was envisioned that two-step pA*f*BPP could be used to map the THC interaction landscape in neuroblastoma cells.

**FIG. 1.**
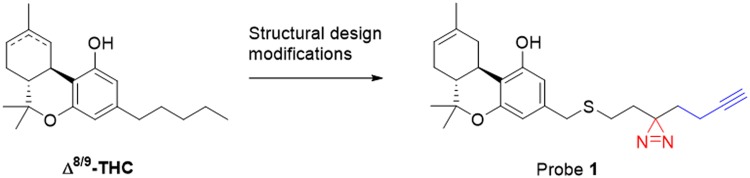
Design of photoaffinity probe 1. The photoreactive diazirine is highlighted in red, the alkyne ligation handle in blue.

To this end, photoaffinity probe 1 (Fig. 1), a Δ^8^-THC analog carrying a diazirine as the photoreactive moiety and a terminal alkyne as the ligation handle, was developed. Probe 1 was synthesized in 14 steps and was found to have high affinity for both cannabinoid receptors (CBRs). The protein interaction landscape of THC was mapped in Neuro2A cells (a fast-growing neuroblastoma cell line with several neuronal properties), in which four putative novel targets of THC were identified.

## Materials and Methods

### Chemistry

#### General remarks

All reactions were performed using air- or flame-dried glassware. Solvents were purchased from Sigma-Aldrich, and dry solvents were analytically dried by storing them for 24 h on activated molecular sieves. Use of dry solvents is mentioned explicitly. Reagents were purchased from Sigma-Aldrich, Acros Organics, and Merck and used without further purification. All moisture sensitive reactions performed under an Ar atmosphere are mentioned explicitly.

^1^H and ^13^C nuclear magnetic resonance (NMR) spectra were recorded on a Bruker AV 400 MHz spectrometer at 400 and 100 MHz, respectively, using CDCl_3_ or CD_3_OD as solvent, unless stated otherwise. Chemical shift values are reported in ppm with TMS or solvent resonance as the internal standard (CDCl_3_/TMS, δ 0.00 for ^1^H [TMS], δ 77.16 for ^13^C [CDCl_3_]; CD_3_OD, δ 3.31 for ^1^H, δ 49.00 for ^13^C). Data are reported as follows: chemical shifts (δ) in ppm, multiplicity (s=singlet, d=doublet, dd=doublet of doublet, ddd=doublet of doublet of doublet, dt=doublet of triplet, t=triplet, td=triplet of doublet, q=quartet, br s=broad singlet, and m=multiplet), coupling constants *J* (Hz), and integration.

High-resolution mass spectra were recorded on a Thermo Scientific LTQ Orbitrap XL. Liquid Chromatography was performed on a Finnigan Surveyor liquid chromatography-mass spectrometry (LC/MS) system, equipped with a C18 column. Thin layer chromatography (TLC) analysis was performed on Merck silica gel 60/Kieselguhr F254, 0.25 mm TLC plates. Compounds were visualized by ultraviolet (UV) irradiation or with a KMnO_4_ stain (K_2_CO_3_ (40 g), KMnO_4_ (6 g), and H_2_O (600 mL)). Molecules shown are drawn using the ChemDraw Professional 16.0.

#### Synthetic procedures to photoaffinity probe 1

**FIG. 2.**
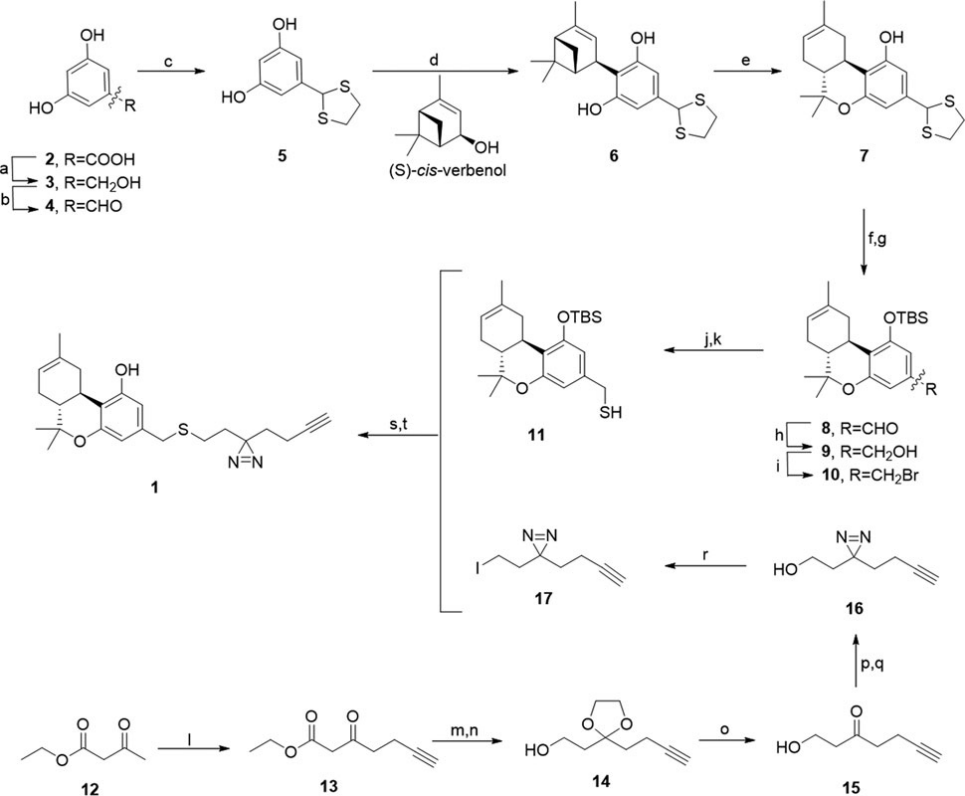
Synthesis of probe 1. Reagents and conditions: **(a)** BH_3_Me_2_S, B(OMe)_3_, THF, rt, 16 h, 99%; **(b)** CrO_3_, H_2_SO_4_, Acetone, 0°C, 10 min, 86%; **(c)** 1,2-ethanedithiol, BF_3_.Et_2_O, THF, rt, 16 h, 99%; **(d)** (s)-*cis*-verbenol, CSA, CHCl_3_, rt, 2 h, 60%; **(e)** BF_3_.Et_2_O, DCM, 0°C-rt, 1.5 h, 62%; **(f)** AgNO_3_, EtOH/H_2_O (10:1), rt, 18 h; **(g)** TBSCl, imidazole, DMF, rt, 3 h, 87% (yield over two steps); **(h)** LiBH_4_, THF, rt, 30 min, 99%; **(i)** CBr_4_, PPh_3_, DCM, rt, 1 h, 98%; **(j)** Thiourea, EtOH, 40°C, 1 h; **(k)** 1 M NaOH (aq.), EtOH, rt, 1 h, 80% (two steps); **(l)** LDA, THF, −40°C, 30 min; then propargyl bromide, 0°C, 1 h, 76%; **(m)** ethylene glycol, TsOH, PhMe, reflux in *Dean-Stark apparatus*, 3 h; **(n)** LiAlH_4_, THF, 0°C, 1 h, 92% (two steps); **(o)** TsOH, 19:1 acetone/H_2_O, 50°C, 2 h, 98%; **(p)** NH_3_ (l), reflux, 5 h; then NH_2_SO_3_H in MeOH, rt, 16 h; **(q)** I_2_, Et_3_N, DCM, 0°C, 83% (two steps); **(r)** I_2_, PPh_3_, imidazole DCM, rt, 1 h, 90%; **(s)** K_2_CO_3_, 2:1 THF/DMF, 30°C, 22 h; **(t)** TBAF, THF, 0°C, 15 min, 84% (two steps). DMF, dimethylformamide; rt, room temperature; THF, tetrahydrofuran.

3,5-Dihydroxybenzyl alcohol **(3)**: A flame-dried 500 mL round bottom flask was charged with a magnetic stirring bar, purged with Ar, and borane-dimethylsulfide complex (18.8 mL, 100 mmol, 3 eq), along with trimethoxy borate (35.6 mL, 313.2 mmol, 4.7 eq) and dry tetrahydrofuran (THF) (30 mL) were added at room temperature (rt) (Fig. 2). The flask was purged with Ar again and 3,5-dihydroxybenzoic acid **2** (10.28 g, 66.6 mmol, 1 eq) in dry THF (50 mL) was added dropwise over 20 min at rt, throughout which rigorous hydrogen gas evolution occurred. The reaction was allowed to stir for 18 h at rt. Upon completion MeOH (100 mL) was added dropwise, throughout which minor hydrogen gas and heat evolution occurred. The solution was filtered through celite, and the filtrate concentrated, and then subsequently coevaporated four more times with MeOH (100 mL each), to give 3,5-dihydroxybenzyl alcohol **3** (9.31 g, 66.3 mmol, 99%) as white/gray amorphous crystals. R_f_: 0.5 (50% EtOAc/pentane). ^1^H NMR (400 MHz, MeOD) δ 6.32 (d, *J*=2.2 Hz, 2H), 6.18 (t, *J*=2.2 Hz, 1H), and 4.47 (s, 2H).

3,5-Dihydroxybenzaldehyde **(4)**: A 500 mL round bottom flask was charged with a magnetic stirring bar, and benzyl alcohol **3** (7.82 g, 55 mmol, 1 eq) and acetone (340 mL) were added. The solution was cooled to 0°C using an ice bath, upon which freshly made 0.9 M Jones reagent (58.5 mL, 52.5 mmol, 1.05 eq) was added dropwise over 10 min. The reaction was stirred for an additional 10 min at 0°C, upon which iPrOH was added (5 mL) and the reaction stirred an additional 5 min, until all yellow color had disappeared, indicating full reduction of residual CrO_3_. The reaction was diluted with Et_2_O (1.5 L) and transferred to a separating funnel. The organic layer was washed with a 1:1 (v/v) solution of sat. NaHCO_3_/brine (150 mL) and then washed successively with brine (8×150 mL). The organic layer was dried over MgSO_4_, and concentrated, to give 3,5-dihydroxybenzaldehyde **4** (6.53 g, 47.3 mmol, 86%) as light brown amorphous crystals. R_f_: 0.4 (40% EtOAc/pentane).^1^H NMR (400 MHz, MeOD) δ 9.77 (s, 1H), 6.79 (d, *J*=2.2 Hz, 2H), and 6.55 (t, *J*=2.2 Hz, 1H).

2-(3,5-Dihydroxyphenyl)-1,3-dithiolane **(5)**: A 500 mL round bottom flask was charged with a magnetic stirring bar, aldehyde **4** (2.9 g, 21 mmol, 1 eq), and purged with Ar. Dry THF (15 mL) was added, and shortly after, dry DCM (180 mL) and 1,2-ethanedithiol (2.65 mL, 31.51 mmol, 1.5 eq) were added. BF_3_.Et_2_O (0.95 mL, 6.93 mmol, 0.33 eq) was added dropwise, upon which the reaction was allowed to stir for 16 h at rt. The reaction was quenched with sat. NaHCO_3_ (200 mL) and transferred to a separating funnel. The pH of the aqueous layer was adjusted to pH 7 with 1 M HCl aq. solution and subsequently extracted with DCM (2×200 mL) and with EtOAc (200 mL). The combined organic layers were dried over MgSO_4_ and concentrated. The resulting brown syrup was dissolved in tBuOMe (20 mL), cooled in an ice bath, and ice-cold hexane (200 mL) was added. The slurry was filtered and the solids washed generously with ice-cold hexane (100 mL) to give **5** (4.56 g, 21 mmol, 99%) as off-white flaky crystals. R_f_: 0.5 (40% EtOAc/pentane). ^1^H NMR (400 MHz, MeOD) δ 6.47 (d, *J*=2.1 Hz, 2H), 6.15 (t, *J*=2.1 Hz, 1H), 5.50 (s, 1H), 3.49–3.42 (m, 2H), and 3.33–3.27 (m, 2H).

5-(1,3-Dithiolan-2-yl)-2-((1R,2S,5S)-4,6,6-trimethylbicyclo[3.1.1]hept-3-en-2-yl)benzene-1,3-diol **(6)**: A 500 mL round bottom flask was charged with a magnetic stirring bar, dithiolane **5** (2.2 g, 10.3 mmol, 1 eq), and purged with Ar. Dry CHCl_3_ (90 mL) was added, along with anhydrous camphorsulfonic acid (0.26 g, 1.03 mmol, 0.1 eq), and the flask purged with Ar again. (S)-*cis*-Verbenol (1.73 g, 11.35 mmol, 1.1 eq, 50% ee) in dry CHCl_3_ (10 mL) was added dropwise, and the reaction allowed to stir at rt for 3 h. Upon completion, the reaction was quenched with an aqueous solution of 1:4 (v/v) sat. NaHCO_3_/brine (100 mL), and transferred to a separating funnel. The pH of the aqueous layer was adjusted to pH 7 with 1 M HCl aq. solution, and subsequently extracted with CHCl_3_ (2×120 mL) and with EtOAc (120 mL). The combined organic layer was dried over MgSO_4_ and concentrated. After concentration, the crude residue (∼4 g) was purified by flash column chromatography (150 g silica), eluting with 10% EtOAc/pentane (8 CV) to give **6** (2.17 g, 6.24 mmol, 60%) as a viscous yellow oil, which forms a foamy amorphous white solid under reduced pressure at rt. R_f_: 0.65 (20% EtOAc/pentane).^1^H NMR (400 MHz, CDCl_3_) δ 6.53 (s, 2H), 5.68 (d, *J*=1.3 Hz, 1H), 5.48 (s, 1H), 3.92 (dd, *J*=5.0, 2.5 Hz, 1H), 3.46–3.43 (m, 2H), 3.33–3.30 (m, 2H), 2.33–2.23 (m, 2H), 2.19–2.16 (m, 1H), 1.85 (s, 3H), 1.50–1.46 (m, 1H), 1.32 (s, 3H), and 0.95 (s, 3H). ^13^C NMR (100 MHz, CDCl_3_) δ 153.2, 140.5, 116.3, 115.0, 55.8, 48.0, 47.1, 40.9, 40.2, 38.1, 28.0, 26.1, 23.9, and 20.6. LC-MS (ESI+) m/z: calculated for C_19_H_25_O_2_S_2_ [M + H]^+^: 349.13, found 349.07.

(6aR,10aR)-3-(1,3-Dithiolan-2-yl)-6,6,9-trimethyl-6a,7,10,10a-tetrahydro-6H-benzo[c]chromen-1-ol **(7)**: A flame-dried 1000 mL round bottom flask was charged with a magnetic stirring bar, bicyclic resorcinol derivative **6** (5.75 g, 16.51 mmol, 1 eq) was added. The flask was purged with Ar, and dry DCM (375 mL) was added, and the solution cooled to 0°C. BF_3_.Et_2_O (4.2 mL, 33 mmol, 2 eq) was added dropwise over 5 min, upon which the reaction was allowed to warm to rt, and was stirred for 1.5 h. Upon completion, the reaction was quenched with an aqueous solution of 1:4 (v/v) sat. NaHCO_3_/brine (450 mL) and was transferred to a separating funnel. The pH of the aqueous layer was adjusted to pH 7 with 1 M HCl aq. solution, and subsequently extracted with CHCl_3_ (2×450 mL). The combined organic layer was dried over MgSO_4_, and concentrated. After concentration, the crude residue (∼6 g) was purified by flash column chromatography (225 g silica), eluting first with 6% EtOAc/pentane (6 CV), then 8% EtOAc/pentane (8 CV) to give **7** (3.39 g, 10.3 mmol, 62%) as a viscous dark yellow oil, which forms a foamy amorphous yellow solid under reduced pressure at rt. R_f_: 0.55 (1% TFA/DCM).^1^H NMR (400 MHz, CDCl_3_) δ 6.56 (s, 1H), 6.48 (d, *J*=1.1 Hz, 1H), 5.48 (s, 1H), 5.41 (d, *J*=3.8 Hz, 1H), 5.26 (s, 1H), 3.49–3.37 (m, 2H), 3.35–3.25 (m, 2H), 3.19 (dd, *J*=16.1, 3.5 Hz, 1H), 2.69 (td, *J*=10.8, 4.6 Hz, 1H), 2.13 (dd, *J*=11.0, 3.5 Hz, 1H), 1.81 (m, 3H), 1.69 (s, 3H), 1.37 (s, 3H), and 1.09 (s, 3H). ^13^C NMR (100 MHz, CDCl_3_) δ 155.3, 155.0, 140.2, 134.8, 119.4, 113.2, 109.9, 106.7, 77.13, 55.8, 44.8, 40.2, 40.1, 35.8, 31.80, 28.0, 27.6, 23.6, and 18.7. LC-MS (ESI+) m/z: calculated for C_19_H_25_O_2_S_2_ [M + H]^+^_:_ 349.13, found 349.07.

(6aR,10aR)-1-((*tert*-Butyldimethylsilyl)oxy)-6,6,9-trimethyl-6a,7,10,10a-tetrahydro-6H-enzo[c]chromene-3-carbaldehyde **(8)**: A 100 mL round bottom flask was charged with a magnetic stirring bar, tricyclic dithiolan **7** (482 mg, 1.38 mmol, 1 eq), and EtOH (40 mL). AgNO_3_ (756 g, 4.43 mmol, 3.2 eq) was added, followed by millipore H_2_O (4 mL), and the flask was sealed with a septum and allowed to stir at rt for 18 h, upon which the reaction was diluted with EtOAc (75 mL), and filtered through celite, washing solids with additional EtOAc (50 mL). The combined filtrate was transferred to a separating funnel and washed with an aqueous solution of 1:1 (v/v) 10% Na_2_SO_3_/Brine (2×50 mL), then with H_2_O (50 mL), and brine (50 mL). The organic layer was dried over MgSO_4_, and concentrated. The crude aldehyde was subsequently dissolved in dry dimethylformamide (DMF) (4 mL) and transferred to a 10 mL round bottom flask, and purged with Ar. *tert*-Butyldimethylsilyl chloride (243 mg, 1.6 mmol, 1.25 eq) was added, followed by imidazole (217 mg, 3.2 mmol, 2.5 eq). The reaction was purged again with Ar and stirred for 3 h at rt. Upon completion, the reaction was quenched with 0.2 M HCl (25 mL), EtOAc (25 mL) was added, and transferred to a separating funnel. The layers were separated, and the aqueous layer was extracted again with EtOAc (25 mL). The combined organic layer was washed with H_2_O (20 mL) and brine (40 mL) and subsequently dried over MgSO_4_, and concentrated. After concentration, the crude residue (∼600 mg) was purified by flash column chromatography (20 g silica), eluting with 10% CHCl_3_ (8 CV)_,_ to give aldehyde **8** (464 mg, 1.20 mmol, 87% over 2 steps) as a clear, viscous oil. R_f_: 0.3 (10% CHCl_3_/pentane). ^1^H NMR (400 MHz, CDCl_3_) δ 9.81 (s, 1H), 6.96 (d, *J*=0.7 Hz, 1H), 6.87 (s, 1H), 5.43 (d, *J*=2.8 Hz, 1H), 3.24 (dd, *J*=16.5, 3.3 Hz, 1H), 2.66 (td, *J*=10.8, 4.3 Hz, 1H), 2.24–2.05 (m, 1H), 1.81 (t, *J*=10.9 Hz, 3H), 1.69 (s, 3H), 1.40 (s, 3H), 1.08 (s, 3H), 1.01 (s, 9H), 0.32 (s, 3H), and 0.18 (s, 3H). ^13^C NMR (100 MHz, CDCl_3_) δ 191.8, 156.0, 155.7, 135.9, 134.7, 124.9, 124.7, 119.5, 114.2, 110.1, 77.3, 45.2, 37.4, 35.7, 33.0, 30.5, 29.8, 28.1, 27.4, 26.0, 25.9, 23.4, 18.4, −3.5, and −4.3.

((6aR,10aR)-1-((*tert*-Butyldimethylsilyl)oxy)-6,6,9-trimethyl-6a,7,10,10a-tetrahydro-6H-benzo[c]hromen-3-yl) methanol **(9)**: A flame-fried 10 mL round bottom flask was charged with a magnetic stirring bar, aldehyde **8** (193 mg, 0.5 mmol, 1 eq), and the flask was purged with Ar. Dry THF was added (2 mL) and the flask cooled to 0°C in an ice water bath. 2 M LiBH_4_ in THF (0.375 mL, 0.75 mmol, 1.5 eq) was added dropwise, upon which the reaction was allowed to warm to rt, and was stirred for 30 min. Upon completion, the reaction was quenched with H_2_O (50 mL) and transferred to a separating funnel. The aqueous layer was extracted with Et_2_O (3×40 mL), and the combined organic layer was dried over MgSO_4_ and concentrated. After concentration, the residue (∼200 mg) was filtered through a short pad of silica (5 g), eluting with CHCl_3,_ to give primary alcohol **9** (192 mg, 495 μmol, 99%) as a turbid, colorless syrup. R_f_: 0.4 (CHCl_3_). ^1^H NMR (400 MHz, CDCl_3_) δ 6.46 (d, *J*=1.2 Hz, 1H), 6.39 (d, *J*=1.2 Hz, 1H), 5.41 (d, *J*=3.6 Hz, 1H), 4.53 (s, 2H), 3.30–3.14 (m, 1H), 2.59 (td, *J*=10.8, 4.2 Hz, 1H), 2.26–2.02 (m, 1H), 1.91–1.57 (m, 4H), 1.68 (s, 3H), 1.37 (s, 3H), 1.07 (s, 3H), 1.00 (s, 9H), 0.27 (s, 3H), and 0.15 (s, 3H). ^13^C NMR (100 MHz, CDCl_3_) δ 155.5, 155.1, 140.4, 135.0, 119.4, 116.7, 109.9, 109.4, 76.9, 65.3, 45.4, 36.1, 32.5, 28.2, 27.5, 26.1, 23.5, 18.4, −3.4, and −4.2.

(((6aR,10aR)-3-(Bromomethyl)-6,6,9-trimethyl-6a,7,10,10a-tetrahydro-6H-benzo[c]chromen-1-yl)oxy) (*tert*-butyl)dimethylsilane **(10)**: A 10 mL round bottom flask was charged with a magnetic stirring bar, 9 (192 mg, 495 μmol, 1 eq), DCM (2.5 mL), and CBr_4_ (172 mg, 519 μmol, 1.05 eq). The flask was cooled to 0°C in an ice water bath, and PPh_3_ (136 mg, 0.519 mmol, 1.05 eq) was added. The reaction was purged with Ar and allowed to come to rt and stirred for 1 h. Upon completion, the reaction was concentrated under reduced pressure, and hexane (1 mL) was added. The resulting slurry was purified by flash column chromatography (15 g silica), eluting first with pentane (6 CV), then with 25% Et_2_O/pentane (6 CV) to give **10** (218 mg, 483 μmol, 98%) as a clear, viscous oil. R_f_: 0.35 (pentane). ^1^H NMR (400 MHz, CDCl_3_) δ 6.48 (d, *J*=1.6 Hz, 1H), 6.40 (d, *J*=1.5 Hz, 1H), 5.41 (d, *J*=2.5 Hz, 1H), 4.41–4.27 (m, 2H), 3.22 (dd, *J*=16.6, 3.6 Hz, 1H), 2.58 (td, *J*=10.8, 4.2 Hz, 1H), 2.20–2.05 (m, 1H), 1.84–1.72 (m, 3H), 1.63 (s, 3H), 1.37 (s, 3H), 1.04 (s, 3H), 1.00 (s, 9H), 0.28 (s, 3H), and 0.15 (s, 3H). ^13^C NMR (100 MHz, CDCl_3_) δ 155.2, 155.0, 136.7, 135.0, 119.4, 117.8, 112.1, 111.6, 76.9, 45.3, 36.0, 33.9, 32.5, 28.2, 27.5, 26.1, 23.5, 18.5, −3.4, and −4.2.

((6aR,10aR)-1-((tert-Butyldimethylsilyl)oxy)-6,6,9-trimethyl-6a,7,10,10a-tetrahydro-6H-benzo[c]chromen-3-yl)methanethiol **(11)**: A 10 mL round bottom flask was charged with a magnetic stirring bar, bromide **10** (31 mg, 68 μmol, 1 eq), and EtOH (1.3 mL). Thiourea (10 mg, 134 μmol, 2 eq) was added, the reaction heated to 40°C in a warm water bath and stirred for 1 h. Upon completion, the reaction was cooled, dry N_2_ gas was bubbled through the reaction for 5 min, and subsequently 1 M NaOH (0.2 mL) was added, and the reaction was stirred another 1 h. Upon completion, the reaction was quenched with 0.1 M HCl (10 mL) and transferred to a separating funnel. The aqueous layer was extracted with Et_2_O (20 mL), and the organic layer was washed with sat. NaHCO_3_ (10 mL), H_2_O (10 mL), brine (10 mL), dried over MgSO_4_, and concentrated. After concentration, the crude residue (∼25 mg) was purified by flash column chromatography (2 g silica), eluting first with pentane (4 CV), then 5% CHCl_3_/pentane (8 CV) to give **11** (22 mg, 54 μmol, 80% over two steps) as a turbid, viscous oil. R_f_: 0.5 (5% CHCl_3_/pentane). ^1^H NMR (400 MHz, CDCl_3_) δ 6.41 (d, *J*=1.7 Hz, 1H), 6.35 (d, *J*=1.7 Hz, 1H), 5.41 (d, *J*=3.8 Hz, 1H), 3.66–3.53 (m, 2H), 3.22 (dd, *J*=16.6, 4.3 Hz, 1H), 2.57 (td, *J*=10.9, 4.3 Hz, 1H), 2.23–2.07 (m, 1H), 1.92–1.68 (m, 3H), 1.68 (s, 3H), 1.36 (s, 3H), 1.07 (s, 3H), 1.00 (s, 9H), 0.27 (s, 3H), and 0.15 (s, 3H). ^13^C NMR (100 MHz, CDCl_3_) δ 155.02, 140.3, 135.0, 119.4, 116.2, 111.2, 110.5, 76.9, 45.4, 36.1, 32.4, 28.9, 28.2, 27.6, 26.1, 23.5, 18.5, −3.4, and −4.2.

Ethyl 3-oxohept-6-ynoate **(13)**: A flame-dried Schlenk tube was charged with a stirring bar and purged multiple times with Ar. Dry THF (30 mL) and then freshly distilled diisopropylamine (9.71 mL, 69.28 mmol) were added, and the solution cooled to −78°C. 1.6 M nBuLi in hexanes (39.38 mL, 63 mmol) was added dropwise, and stirred for 15 min. The generated LDA solution (0.8 M by titration, 73 mL, 2.12 eq) was transferred via cannula to a flame-dried 250 mL round bottom flask. The flask was cooled to −40°C, upon which ethyl acetoacetate 12 (3.47 mL, 27.5 mmol, 1 eq) in dry THF (25 mL) was added dropwise. The reaction was stirred for 30 min, upon which propargyl bromide (80% in toluene, 3 mL, 28 mmol, 1.01 eq) was added dropwise, and the reaction was allowed to warm to 0°C and stirred for 1 h. Upon completion, the reaction was quenched with 0.5 M HCl (200 mL) and transferred to a separating funnel. The aqueous layer was extracted with Et_2_O (2×200 mL). The combined organic layers were washed with brine (100 mL), dried over MgSO_4_, and concentrated. The resulting amber syrup (4.80 g) was purified by fractional distillation (118°C, 15 mBar) to give **13** (3.49 g, 20.8 mmol, 76%) as a clear oil. R_f_: 0.4 (50% CHCl_3_/hexane). ^1^H NMR (400 MHz, CDCl_3_) δ 4.20 (q, *J*=7.1 Hz, 2H), 3.47 (s, 2H), 2.82 (t, *J*=7.2 Hz, 2H), 2.53–2.43 (m, 2H), 1.97 (t, *J*=2.7 Hz, 1H), and 1.29 (t, *J*=7.1 Hz, 3H). ^13^C NMR (100 MHz, CDCl_3_) δ 200.7, 167.0, 90.2, 82.6, 69.1, 61.6, 49.3, 41.7, 14.2, and 12.9.

2-(2-(But-3-yn-1-yl)-1,3-dioxolan-2-yl)ethan-1-ol **(14)**: A 50 mL round bottom flask was charged with a magnetic stirring bar, equipped with a Dean-Stark apparatus, and purged with Ar. Thirteen (383 mg, 2.27 mmol, 1 eq) in toluene (35 mL) was added, along with ethylene glycol (211 mg, 1.5 eq), followed by *para*-toluenesulfonic acid (39 mg, 0.23 mmol, 0.1 eq), and the reaction heated to reflux for 3 h. Upon completion, the reaction was quenched with sat. NaHCO_3_ (25 mL), diluted with EtOAc (25 mL), and transferred to a separating funnel. The organic layer was washed with H_2_O (50 mL), brine (50 mL), dried over MgSO_4_, and concentrated. The crude ester was dissolved in dry THF (10 mL) and added dropwise to a flame-dried 25 mL round bottom flask previously cooled to 0°C, purged with Ar, and containing LiAlH_4_ (150 mg, 3.97 mmol, 1.75 eq) suspended in dry THF (10 mL). Upon addition, the reaction was allowed to warm to rt and stirred for 1 h. Upon completion, the reaction was quenched with 10 mL EtOAc, stirred for 5 min, and aq. 10 wt.% Rochelle's salt (50 mL) was added and stirred for an additional 10 min. The reaction was transferred to a separating funnel, upon which the organic layer was washed with brine (30 mL), dried over MgSO4, and concentrated. After concentration, the crude residue was filtered through a short pad of silica (∼10 g), eluting with CHCl_3_, to give **14** (357 mg, 2.09 mmol, 92% over two steps), as a pale yellow oil. R_f_: 0.35 (50% CHCl_3_/hexane). ^1^H NMR (400 MHz, CDCl_3_) δ 4.00 (m, 4H), 3.75 (t, *J*=5.7 Hz, 2H), 2.73 (s, 1H), 2.42–2.21 (m, 2H), and 2.05–1.79 (m, 5H). ^13^C NMR (100 MHz, CDCl_3_) δ 111.0, 84.0, 68.3, 65.0, 58.6, 38.4, 36.0, and 13.1.

1-Hydroxyhept-6-yn-3-one **(15)**: A 25 mL round bottom flask was charged with a magnetic stirring bar, and **14** (357 mg, 2.09 mmol, 1 eq) in acetone (9.5 mL) was added, followed by *para*-toluenesulfonic acid (99 mg, 0.52 mmol, 0.25 eq), and millipore H_2_O (0.5 mL), and the reaction was heated to 50°C for 2 h. Upon completion, the reaction was quenched with sat. NaHCO_3_ (10 mL), diluted with EtOAc (30 mL), and transferred to a separating funnel. The organic layer was washed with brine (20 mL), dried over MgSO_4_, and concentrated. After concentration, the crude residue was filtered through a short pad of silica (∼15 g), eluting with CHCl_3_, to give **15** (258 mg, 2.05 mmol, 98%) as a pale yellow oil. R_f_: 0.25 (CHCl_3_). ^1^H NMR (400 MHz, CDCl_3_) δ 3.87 (t, *J*=5.5 Hz, 2H), 2.71 (dd, *J*=9.1, 5.5 Hz, 4H), 2.55–2.40 (m, 3H), and 1.97 (t, *J*=2.5 Hz, 1H). ^13^C NMR (100 MHz, CDCl_3_) δ 209.1, 82.9, 69.0, 57.8, 44.7, 41.9, and 12.9.

2-(3-(But-3-yn-1-yl)-3H-diazirin-3-yl)ethan-1-ol **(16)**: A 50 mL amber three-necked flask was charged with a magnetic stirring bar, purged with Ar, and cooled to −50°C in an acetone-dry ice bath. NH_3_ gas (5 mL) was condensed into the flask using a dry ice condenser, upon which **15** (255 mg, 2.04 mmol) in dry DCM (1 mL) was added dropwise. The reaction was allowed to warm to −40°C and was stirred at reflux for 5 h, upon which hydroxylamine-*O*-sulfonic acid (425 mg, 3.76 mmol, 1.83 eq) in dry MeOH (1 mL) was added dropwise. The reaction was kept at reflux for an additional 1 h, and then allowed to warm to rt over 16 h. Dry N_2_ was subsequently bubbled through the reaction, allowing all excess NH_3_ to evaporate, the reaction was filtered over celite, and the filter cake was washed with dry MeOH (40 mL). The filtrate was concentrated under reduced pressure, and the crude diaziridine residue redissolved in DCM (2 mL) and transferred to a 10 mL round bottom flask, purged with Ar, and cooled to 0°C in an ice bath. Dry Et_3_N (0.5 mL) was added, and a solution of I_2_ (500 mg) in DCM (8 mL) was added dropwise over 1 h until a brown/red color persisted for at least 0.5 h. Upon completion, the reaction was quenched with 1 M HCl (3 mL), and diluted with EtOAc (40 mL) and transferred to a separating funnel. The organic layer was washed with aq. 10 wt.%% (2×20 mL), brine (20 mL), dried over MgSO_4_, and concentrated. After concentration, the crude residue (∼250 mg) was purified by flash column chromatography (10 g silica), eluting with 75% CHCl_3_/pentane (2 CV), 80% CHCl_3_/pentane (4 CV), then CHCl_3_ (4 CV) to give **16** (234 mg, 1.69 mmol, 83% over two steps) as a dark yellow oil. R_f_: 0.4 (CHCl_3_). ^1^H NMR (400 MHz, CDCl_3_) δ 3.49 (t, *J*=6.2 Hz, 2H), 2.14–1.95 (m, 3H), 1.85 (br s, 1H), and 1.74–1.63 (m, 4H). ^13^C NMR (100 MHz, CDCl_3_) δ 82.9, 69.4, 57.4, 35.6, 32.7, 26.7, and 13.3.

3-(But-3-yn-1-yl)-3-(2-iodoethyl)-3H-diazirine **(17)**: A 25 mL amber flask was charged with a magnetic stirring bar, and **16** (234 mg, 1.69 mmol, 1 eq). DCM (7.5 mL) was added. The flask was cooled to 0°C in an ice bath, and imidazole (345 mg, 5.07 mmol, 3 eq), was added, followed by I_2_ (515 mg, 2.03 mmol, 1.2 eq) and PPh_3_ (488 mg, 1.86 mmol, 1.1 eq). The reaction was purged with Ar, allowed to come to rt, and stirred for 1 h. Upon completion, the reaction was quenched with aq. 10 wt.% Na_2_S_2_O_3_ (10 mL) and transferred to a separating funnel. The aqueous layer was extracted with CHCl_3_ (3×20 mL). The combined organic layer was dried over MgSO_4_ and concentrated. After concentration, the crude residue (∼500 mg) was purified by flash column chromatography (25 g silica), eluting with pentane (6 CV), then 5% Et_2_O/pentane (6 CV) to give **17** (378 mg, 1.52 mmol, 90%) as a clear oil. R_f_: 0.25 (pentane). ^1^H NMR (400 MHz, CDCl_3_) δ 2.90 (t, *J*=7.6 Hz, 2H), 2.13 (t, *J*=7.6 Hz, 2H), 2.07–1.96 (m, 3H), and 1.69 (t, *J*=7.1 Hz, 2H). ^13^C NMR (100 MHz, CDCl_3_) δ 82.6, 77.4, 69.6, 37.7, 32.0, 28.8, 13.4, and −3.9.

(6aR,10aR)-3-(((2-(3-(But-3-yn-1-yl)-3H-diazirin-3-yl)ethyl)thio)methyl)-6,6,9-trimethyl-6a,7,10,10a-tetrahydro-6H-benzo[c]chromen-1-ol **(1)**: A 10 mL round bottom flask was charged with a magnetic stirring bar, tricyclic probe precursor **11** (20 mg, 49 μmol, 1 eq), and THF (0.5 mL), and the reaction cooled to 0°C in an ice water bath. Minimalist linker **17** (19 mg, 75 μmol, 1.53 eq) in THF (0.5 mL) was added, followed by anhydrous K_2_CO_3_ (13.5 mg, 98 μmol, 2 eq), and DMF (0.5 mL). The reaction was purged with Ar again, allowed to come to rt, then warmed to 30°C, and stirred for 22 h. Upon completion, H_2_O (10 mL) was added, and the reaction was transferred to a separating funnel. The aqueous layer was extracted with CHCl_3_ (2×10 mL), and the combined organic layer was washed with brine (10 mL), dried over MgSO_4_, and concentrated. The crude silyl ether was subsequently dissolved in THF (0.5 mL) and transferred to a 10 mL round bottom flask, purged with Ar, and cooled to 0°C in an ice water bath. 1 M TBAF in THF (98 μL, 98 μmol, 2 eq) was added, and the reaction stirred for 15 min at 0°C. Upon completion, the reaction was quenched with H_2_O (10 mL), and transferred to a separating funnel. The aqueous layer was extracted with Et_2_O (10 mL) and the organic layer washed with brine (10 mL), dried over MgSO_4_, and concentrated. After concentration, the crude residue (∼25 mg) was purified by flash column chromatography (2 g silica), eluting first with 20% CHCl_3_/pentane (4 CV), 40% CHCl_3_/pentane (4 CV), and then 50% CHCl_3_/pentane (4 CV) to give **probe 1** (17 mg, 41 μmol, 84% over two steps) as a clear, viscous oil. R_f_: 0.3 (50% CHCl_3_/pentane). ^1^H NMR (400 MHz, CDCl_3_) δ 6.34 (d, *J*=1.2 Hz, 1H), 6.26 (s, *J*=1.6 Hz, 1H), 5.43 (d, *J*=3.8 Hz, 1H), 4.87 (s, 1H), 3.52 (s, 2H), 3.19 (dd, *J*=16.0, 4.0 Hz, 1H), 2.70 (td, *J*=10.7, 4.5 Hz, 1H), 2.25 (m, 3H), 1.99 (m, 3H), 1.81 (t, *J*=8.9 Hz, 3H), 1.70 (s, 3H), 1.62 (dd, *J*=14.7, 7.5 Hz, 5H), 1.38 (s, 3H), and 1.10 (s, 3H). ^13^C NMR (100 MHz, CDCl_3_) δ 155.4, 155.2, 137.7, 134.8, 119.5, 111.0, 107.7, 77.1, 69.4, 44.9, 36.1, 36.0, 33.0, 32.30, 31.8, 29.9, 28.0, 27.7, 25.7, 23.6, 18.6, and 13.4. LC-MS purity found >95%. High resolution mass spectrometry (HRMS) (ESI+) m/z: calculated for C_24_H_31_N_2_O_2_S [M + H]^+^: 411.2101, found 411.2100.

### Biology

#### General remarks

All common reagents were purchased from commercial sources and used as received. Probe 1 was synthesized as described above, Δ^9^-THC, Δ^8^-THC and CY5-N_3_ were synthesized according to previously published procedures^29,30^ and biotin-N_3_ was purchased from Bio-Connect Life Sciences. [^3^H]CP55940 (specific activity 141.2 Ci/mmol) and GF-B/GF-C filters were purchased from Perkin Elmer (Waltham, MA). The CHO-K1 CNR1 and CNR2 cell lines (catalog numbers 93-0959C2 and 93-0706C2, respectively) were obtained from DiscoveRx. Cell culture plates were purchased from Sarstedt.

Cannabinoid receptor ligands CP55940 and AM630 were obtained from Sigma Aldrich (St. Louis, MO), and rimonabant was obtained from F. Hoffmann-La Roche Ltd. (Basel, Switzerland). Reagents used for the pulldown procedure are: avidin-agarose from egg white (50% glycerol suspension from Sigma Aldrich), 10× phosphate buffered saline (PBS) (proteomics grade, Sigma Aldrich) and Trypsin, sequencing grade (Promega). The CaproBox™ was kindly provided by Caprotec Bioanalytics GmbH, Berlin. All buffers and solutions were prepared using Millipore water (deionized using a MilliQ A10 Biocel™, with a 0.22 μm filter) and analytical grade reagents and solvents. Buffers are prepared at rt and stored at 4°C, unless stated otherwise.

#### Cell culture and membrane preparation

CHOK1hCB_1__bgal and CHOK1hCB_2__bgal (source; DiscoveRx, Fremont, CA) were cultured in Ham's F12 Nutrient Mixture supplemented with 10% fetal calf serum, 1 mM glutamine, 50 μg/mL penicillin, 50 μg/mL streptomycin, 300 mg/mL hygromycin, and 800 μg/mL geneticin in a humidified atmosphere at 37°C and 5% CO_2_. Cells were subcultured twice a week at a ratio of 1:20 on 10-cm ø plates by trypsinization. For membrane preparation, the cells were subcultured 1:10 and transferred to large 15 cm diameter plates. Next, the cells were detached by scraping them into 5 mL PBS and collected and centrifuged at 1000 g for 5 min. Pellets derived from 30 plates were added together and resuspended in 20 mL ice-cold buffer (50 mM Tris-HCl, 5 mM MgCl_2_, pH 7.4). An UltraThurrax homogenizer was used to homogenize the cell suspension. Membranes and the cytosolic fraction were separated by ultracentrifugation (100,000 g, with a Ti-70 rotor in a Beckham Coulter Ultracentrifuge) at 4°C for 20 min. The supernatant was discarded and the pellet was resuspended in 10 mL of the same buffer and the homogenization and centrifugation steps were repeated. Supernatant was discarded and the pellet was resuspended in 5 mL buffer. Aliquots of 200 μL were frozen at −80°C until further use. Protein concentration was determined using the BCA method.^31^

#### [^3^H]CP55940 displacement assay

The affinity of probe 1 on CBRs was determined on membrane fractions of CB_1_R- or CB_2_R overexpressing CHO cells, as described previously.^13^ Membrane aliquots containing 5 μg (CHOK1hCB_1__bgal) or 1 μg (CHOK1hCB_2__bgal) of membrane protein in 100 μL assay buffer (50 mM Tris–HCl, 5 mM MgCl_2_, 0.1% BSA, pH 7.4) were incubated at 30°C for 1 h, in presence of 3.5 nM (CHOK1hCB_1__bgal) or 1.5 nM [^3^H]CP55940 (CHOK1hCB_2__bgal). Nonspecific binding was determined in the presence of 10 μM SR141716A (CHOK1hCB_1__bgal) or 10 μM AM630 (CHOK1hCB_2__bgal). Incubation was terminated by rapid filtration performed on GF/C filters (Whatman International, Maidstone, United Kingdom), presoaked for 30 min with 0.25% polyethyleneimine (PEI), using a Brandel harvester (Brandel, Gaithersburg, MD). Filter-bound radioactivity was determined by scintillation spectrometry using a Tri-Carb 2900 TR liquid scintillation counter (Perkin Elmer, Boston, MA).

### Data analysis

Graphs and statistics were performed with GraphPad Prism 7, using the results of three independent experiments performed in duplicate. The nonlinear regression analysis for one site—Fit K_i_ (constrains: top=100 and bottom=0) was used to obtain logK_i_ values, which are provided by Prism by direct application of the Cheng–Prusoff equation^32^: K_i_=IC_50_/(1 + ([L]/K_D_)), in which [L] is the exact concentration of [^3^H]CP55940 determined per experiment (i.e., ∼3.5 or ∼1.5 nM) and K_D_=0.10 (CB_1_R) or 0.33 (CB_2_R) nM of [^3^H]CP55940.

### Two-step photoaffinity labeling, gel-based analysis

Wild type (WT)CHO, CB_1_R, and CB_2_R membrane aliquots were diluted to 2 μg/μL and homogenized for 20 sec with a Heidolph Silent crusher at 25,000 rpm, and benzonase was added (1:10,000 dilution from working stock of 2,500,000 U/mL, assay concentration: 250 U/mL). Eighteen microliters of protein was added per well of a 96-well flat bottom plate and 20 μM CP55940 or MilliQ water with the same% of dimethylsulfoxide (DMSO) was added, but the sample without UV was kept in an Eppendorf tube protected with alumina foil. After incubation of 30 min at rt, 2 μM LEI121 or probe 1, or MilliQ water with the same% of DMSO was added, and the protein was again incubated for 30 min at rt. The samples were then diluted with 30 μL 50 mM Hepes buffer and irradiated for 5 min with CaproBox, preset at 350 nm and cooled during irradiation. The ligation reaction was then performed with 5 μL click master mix per sample (0.455 mM CuSO_4_, 2.73 mM NaAsc, 0.09 mM THPTA, 3.6 μM Cy5-N_3_). The click mix is prepared as follows: 2.5 μL 10 mM CuSO_4_ and 1.5 μL 100 mM NaAsc were mixed together until the copper is fully reduced (visible by the change from the rusty brown color to bright yellow), then 0.5 μL 10 mM THPTA and 0.5 μL 0.4 mM CY5-N_3_ were added. After incubation in the dark for 1 h, the protein was denatured with 18 μL 4×Laemmli sample buffer, and the samples were resolved on a 12.5% acrylamide gel (12 μL per sample per well). Bio-Rad ImageLab was used for gel analysis and quantification.

### Chemoproteomic profiling of THC protein targets

Neuro2A cells were cultured at 37°C with 7% CO_2_ in DMEM supplemented with 10% New Born Calf serum, 10% fetal calf serum, 1 mM glutamine, 50 μg/mL penicillin, and 50 μg/mL streptomycin and passaged twice a week. Cells were washed with PBS, then pretreated in PBS, containing 1 mM MgCl_2_ and 1 mM CaCl_2_, with or without 10 μM THC, for 30 min at 37°C. Then, 1 or 10 μM probe 1 (or the same amount of DMSO for the untreated control) was added (final concentration in a total volume of 3 mL) and incubated for 30 min at 37°C. The solution was removed from the cells and replaced by 1.5 mL PBS containing 1 mM MgCl_2_ and 1 mM CaCl_2_, then the plates were immediately irradiated (except the No UV control) with CaproBox (350 nm) for 5 min, and the cells were harvested by scraping.

The cells were pelleted (10 min, 1200 g, 4°C), supernatant removed, and resuspended in 250 μL 50 mM Hepes buffer. The cells were destroyed with the Heidolph Silent Crusher (20 sec, 25,000 rpm). Samples were sonicated for 10×2.5 sec with 0.5 sec interval (using a probe sonicator from Branson, Digital Sonifier) and 2 μL of 10% sodium dodecyl sulfate (SDS) was added. If samples were frozen at −80°C before continuation of the experiment, the samples were sonicated again for 10×0.5 sec with 0.5 sec interval using a probe sonicator. The protein content was quantified using Bradford^33^ and the experiment was continued using the same amount of protein for each sample. Sample volumes were adjusted to 400 μL with 50 mM Hepes buffer, then the ligation reaction with biotin-N_3_ was performed with 43,7 μL click mix per sample (1.25 mM CuSO_4_, 7.5 mM NaAsc, 0.25 mM THPTA, 22.5 μM biotin-N_3_) for 1 h at rt in the dark. For step-by-step preparation of the click reagents, mix 21.85 μL 25 mm CuSO_4_, 13.15 μL 250 mM NaAsc, 4.37 μL 25 mM THPTA, and 4.37 μL 2.25 mM biotin-N_3_ in this order.

To remove all click components, the protein was precipitated by the addition of 666 μL MeOH, 166 μL CHCl_3_, and 300 μL MilliQ and centrifuged at 20,238 g for 10 min. The supernatant was removed and the pellet was resuspended in 600 μL MeOH using sonication (6×0.5 sec, interval 0.5 sec). The protein was pelleted at 20,238 g for 10 min and the supernatant removed. The protein was then denatured in 15 min at rt with 500 μL 1% SDS containing 25 mM NH_4_HCO_3_, followed by reduction (65°C, 15 min, 700 rpm shaking) using 5 μL 1 M DTT per sample. Samples were cooled to rt before alkylation with 40 μL 0.5 M IAA per sample for 30 min at rt in the dark. One hundred forty microliters of 10% SDS was added per sample, and each sample was added to 6 mL PBS containing 50 μL avidin beads (prewashed with PBS 3×, pelleting at 2000 g for 2 min), and incubated for 2 h at rt while rotating. Beads were pelleted (2000 g, 2 min) and washed with PBS with 0.5% SDS (1×) and with PBS (3×).

On-bead digest of peptides was performed overnight at 37°C, at 1000 rpm with digestion buffer (250 μL per sample, recipe: 300 μL 1 M Tris, 300 μL 1 M NaCl, 3 μL of 1 M CaCl_2_, 60 μL ACN, 3 μL 0.5 μg/μL Trypsin and 2334 μL MilliQ). Samples were quenched with 12.5 μL formic acid (FA) and beads were removed using a Biospin column (600 g, 2 min). Samples were added on C18 StageTips (conditioned with 50 μL MeOH, then 50 μL of 0.5% (v/v) FA in 80% (v/v) ACN/MilliQ (solution B), then 50 μL 0.5% (v/v) FA in MilliQ (solution A), each conditioning step was performed using centrifugation for 2 min at 600 g) by spinning for 15 min at 800 g, then washed with solution A for 10 min at 800 g, and eluted with solution B for 5 min at 800 g into low-binding Eppendorf tubes. Samples were evaporated using an Eppendorf SpeedVac (Eppendorf Concentrator Plus 5301) and 50 μL of LC/MS solution was added (recipe for 2 mL: 1900 μL MilliQ, 60 μL ACN, 2 μL FA, 40 μL of 1 nmol/μL yeast enolase stock). Samples were measured using a NanoACQUITY UPLC System coupled to a SYNAPT G2-Si high-definition mass spectrometer (Waters). The peptides were separated using an analytical column (HSS-T3 C18 1.8 μM, 75 μM×250 mm, Waters) with a concave gradient (5–40% ACN in H_2_O with 0.1% FA). [Glu^1^]-fibrinopeptide B was used as lock mass. Mass spectra were acquired using the UDMS^e^ method.^34^ The mass range was set from 50 to 2000 Da with a scan time of 0.6 sec in positive, resolution mode. The collision energy was set to 4 V in the trap cell for low-energy MS mode. For the elevated energy scan, the transfer cell collision energy was ramped using drift time-specific collision energies.

Raw data were processed using Progenesis QI for Proteomics (3.0, Waters), with lock mass correction (7,858,426 Da) and a database search was performed against the proteomic database of *Mus musculus*, with trypsin as digestion reagent, max two missed cleavages, carbamidomethyl C as a fixed modification, oxidation M as a variable modification, and FDR set to 1%. Relative quantitation using Hi-3 was performed after filtering the peptides on score (cutoff 5).

### Data analysis

The average normalized abundance of proteins in sample replicates of two independent experiments was used to calculate the ratio of proteins in the probe-treated sample and the “No UV” sample, to determine the level of enrichment by UV-irradiation (Fig. 5A). Protein targets that were enriched >2×by probe 1 are shown in Supplementary Table S1. Proteins that were <2-fold enriched and highly abundant (>20%) in the “CRAPome” database^35^ www.crapome.org/, version 1.1) were excluded from further analysis. Gene ontology data of the ∼150 resulting putative probe targets (Fig. 5B, C) were derived using the DAVID Bioinformatics Database (https://david.ncifcrf.gov/home.jsp, version 6.8).

In THC competition experiments, the normalized abundance of proteins in sample replicates of three independent experiments was used to calculate the ratio of proteins in THC-pretreated samples over probe-treated samples. The average of the mean ratios of the triplicate samples of each independent experiment was used to calculate the effect of THC (as fold change), and a Student's *t*-test was used to determine whether the fold change was significantly lower than 1, indicating a significant reduction of the abundance of that particular protein in the THC-treated samples (Fig. 5D). A *p*-value less than 0.05 was considered statistically significant. Proteins that showed <50% inhibition (Supplementary Table S2) were excluded from gene ontology analysis. This analysis yielded one putative protein target of Δ^9^-THC and three putative targets of Δ^8^-THC (Fig. 5A, B, [red dots] and E). Gene ontology and KEGG pathway analysis of the resulting putative protein targets was derived using the DAVID Bioinformatics software (https://david.ncifcrf.gov/home.jsp, version 6.8). In addition, it was investigated whether these proteins are associated with pathophysiologies or diseases using the Online Mendelian Inheritance in Man (OMIM) database (www.omim.org/, September 2017).

## Results and Discussion

### Synthesis of photoaffinity probe 1

To identify the best position in THC to introduce the photoreactive group and the ligation tag, an analysis of previously reported structure-activity relationship data of THC analogs was conducted.^36^ This led to the design of probe 1, which contains a diazirine and ligation handle on the alkyl side chain of THC. An advantage of this design is the direct coupling of the bifunctional side chain as “minimalist linker.”^37^

The synthesis of probe 1 commenced with reduction of commercially available 3,5-dihydroxybenzoic acid 2 to corresponding benzyl alcohol 3 in near-quantitative yield, using dimethyl sulfide complex of borane, along with co-reagent trimethoxyborate (Fig. 2).^38^ Benzyl alcohol 3 was oxidized to aldehyde 4 using a stoichiometric amount of Jones reagent, which prevented overoxidation to the benzoid acid.^39^ Protection of the aldehyde was performed under Lewis acidic conditions, which resulted in 1,3-dithiolane 5 in excellent yield. Electrophilic aromatic substitution of resorcinol derivative 5 under acidic conditions with the commercially available chiral monoterpene (S)-*cis*-verbenol yielded bicyclic intermediate 6 in moderate yield.

The tricyclic intermediate 7 was obtained in moderate yield by ring-closing rearrangement of bicyclic dithiolane 6, due to the generation of side products. Δ^8^-THC was synthesized in two steps from olivetol and (S)-*cis*-verbenol using the same procedures, in a similar yield and comparable to literature.^30^ Intermediate 7 was deprotected by Ag(I) salts, using a AgNO_3_/wet EtOH system.^40^ Overoxidation of the resulting aldehyde to the equivalent benzoic acid was prevented using a modified workup, comprised additional washing steps with 10 wt.% Na_2_SO_3_ (aq.), on top of the sole filtration step described in the literature.^40^ The resulting aldehyde was not isolated but subjected directly to phenol protection with TBS ether, to yield aldehyde 8 in excellent yield over 2 steps. Reduction of 8 to benzyl alcohol 9 with LiBH_4_ proceeded with near-quantitative yield, and a subsequent Appel reaction afforded benzyl bromide 10 in excellent yield. Benzyl mercaptan 11 was obtained by substitution of the bromide by thiourea, followed by cleavage of the amidine moiety from the sulfur atom with NaOH (aq).

The synthesis of minimalist linker 17 started with the functionalization of commercially available ethyl acetoacetate 12 to propargyl ketoester 13 via generation of the dienolate under strongly basic conditions, followed by regiospecific electrophilic attack by propargyl bromide.^37^ Ketoester 13 was then protected with ethylene glycol to the corresponding ketal, with azeotropic removal of water under acidic conditions, followed by direct reduction of the ester group with LiAlH_4_, afforded corresponding alcohol 14 with excellent yield over 2 steps.^41^ Deprotection of ketal 14 afforded ketone 15 in a near-quantitative yield, which was next functionalized by refluxing in liquid NH_3_, followed by addition of hydroxylamine-*O*-sulfonic acid. The resulting crude diaziridine was subsequently oxidized to diazirine 16 using molecular iodine in mild basic conditions and was obtained in high yield over 2 steps. Sixteen then underwent a modified Appel reaction to generate minimalist linker 17 as alkyl iodide, in excellent yield.

Finally, minimalist linker 17 was coupled overnight at 30°C to resorcinol mercaptan 11 using K_2_CO_3_ in a 2:1 THF/DMF solvent system and the crude sulfide underwent rapid TBS ether deprotection in the presence of TBAF, affording target probe 1 in high yield over two steps. Overall, probe 1 was synthesized from commercially available 3,5-dihydroxybenzoic acid 2 in 14 steps, with a total yield of 18%.

### CBR binding affinity of probe 1

**FIG. 3.**
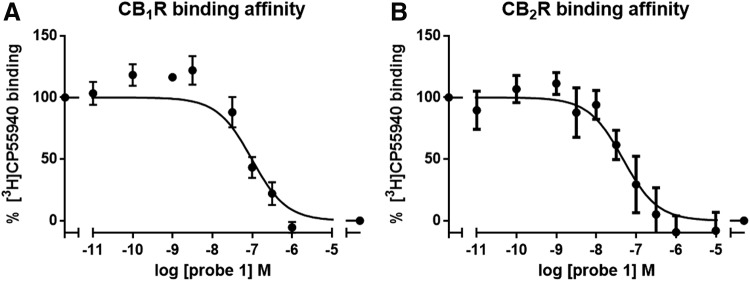
CBR binding affinity of probe 1. Binding affinity of probe 1 was measured on membrane fractions of **(A)** CB_1_R- or **(B)** CB_2_R-overexpressing CHO cells, using previously described [^3^H]CP55940 displacement assays.^13^ CBR, cannabinoid receptor.

To test the affinity of probe 1 on both the CB_1_R and CB_2_R, a [^3^H]CP55940 displacement assay was used (Fig. 3). Probe 1 bound to the CB_1_R and CB_2_R with a pK_i_ value of 8.5±0.1 and 8.0±0.4, respectively, which is similar as previously reported for Δ^9^-THC (pK_i_=8.5±0.1 and 8.2±0.2, respectively) and Δ^8^-THC (pK_i_=7.4±0.1 and 7.4±0.2, respectively).^13,14^

### Two-step photoaffinity labeling of CB_1_R and CB_2_R

**FIG. 4.**
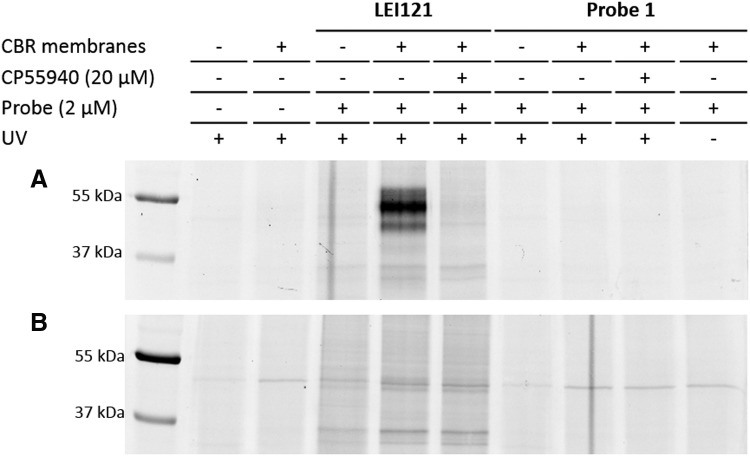
Gel-based analysis of two-step photoaffinity labeling efficiency of probe 1. Probe 1 was not able to covalently label the CBRs in membranes from **(A)** CB_2_R- or **(B)** CB_1_R-overexpressing CHO cells, whereas a CB_2_R-selective probe (LEI121)^28^ specifically labeled CB_2_R **(A)**.

The ability of probe 1 to label CBRs in membranes of CB_2_R- or CB_1_R-overexpressing CHO cells was tested using a two-step photoaffinity labeling assay for gel-based imaging as previously reported.^28^ Probe 1 at a concentration of 2 μM, which is more than sufficient to fully occupy the binding site of the receptors, did not label either one of the CBRs (Fig. 4). Of note, positive control LEI121, a CB_2_R-selective photoaffinity probe previously reported,^28^ did show profound labeling of CB_2_R. This may indicate that the diazirine of probe 1, positioned on the “flexible” alkylic side chain, is not in close proximity to the amino acid residues in the binding site of CB_1_R and CB_2_R to form a covalent bond with the protein. This observation is in line with previous results showing that the position of the photoreactive diazirine on the scaffold of CBR ligands is essential to capture the CBR.^28^

### Chemoproteomic profiling of THC protein targets using probe 1

**FIG. 5.**
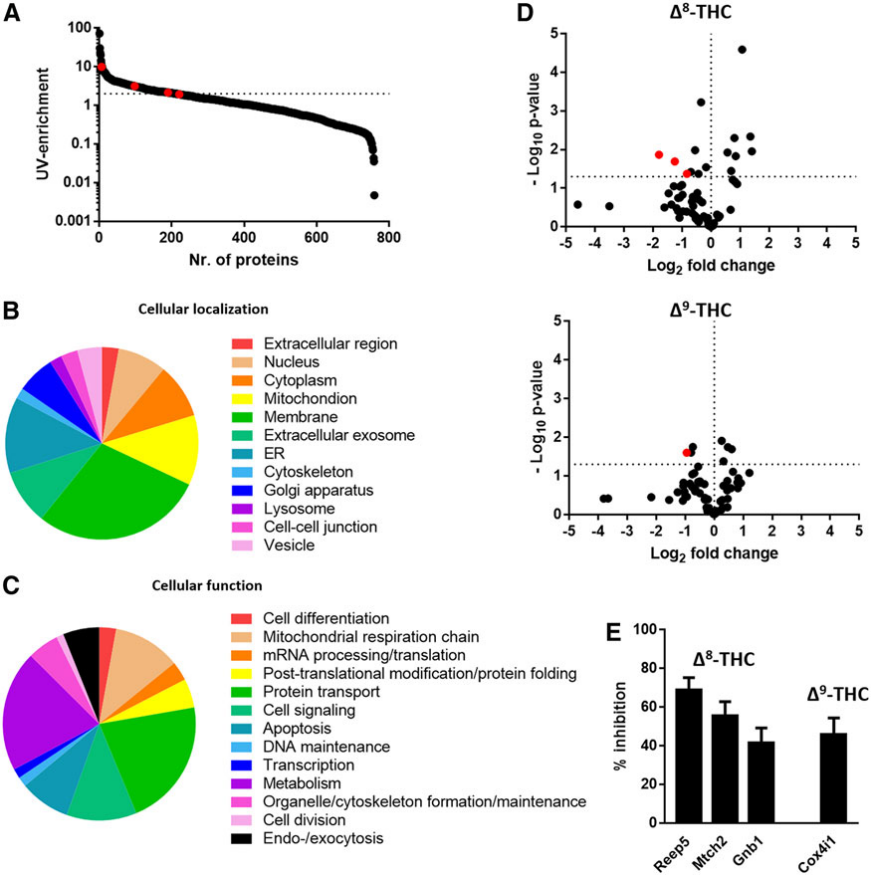
Proteomic analysis of proteins targeted by probe 1. **(A)** Representative plot showing the level of enrichment by probe 1 after UV-irradiation. (**B, C)** Pie charts showing gene ontology analysis of the cellular location **(B)** and cellular function **(C)** of probe targets identified in two independent experiments performed in duplicate. **(D)** Volcano plots showing the fold change in abundance of probe targets after pretreatment with either Δ^8^-THC and Δ^9^-THC (ratio THC pretreated samples over noncompetition samples). Proteins of which the abundance were >40% lowered by THC (*p*-value <0.05) are shown in red. Statistics performed was an unpaired Student's *t*-test. **(E)** 3 and 1 putative protein targets of Δ^8^-THC and Δ^9^-THC, respectively, were identified. Data shown are the mean±SEM of three independent experiments performed in triplicate. THC, Δ^9^-Tetrahydrocannabinol. SEM, standard error of the mean; UV, ultraviolet.

The ability of probe 1 as a chemical tool to identify additional, non-CBR, protein targets of THC was evaluated next. Live Neuro2A cells (a fast-growing neuroblastome cell line with neuronal properties and therefore a suitable test system) were incubated with probe 1 (10 μM). Vehicle-treated and nonirradiated cells were used as control. Ligation with biotin-N_3_ for affinity enrichment on avidin agarose beads enabled identification of nearly 800 proteins by mass spectrometry-based proteomics (Fig. 5A).

Nearly 200 proteins were more than twofold enriched by probe 1 compared to the untreated control, of which ∼50 proteins were also found in the “CRAPome” database (Contaminant Repository for Affinity Purification).^35^ The CRAPome database constitutes a list of frequently identified proteins (e.g., ribosomal proteins or histones) in photoaffinity labeling experiments regardless of the type of probe. These CRAPome proteins can, therefore, be considered as false positives, suggesting that nearly 150 unique probe targets were identified. Gene ontology analysis revealed that protein targets of probe 1 are mostly located in the endoplasmic reticulum, mitochondria and membranes or in the cytoplasm (Fig. 5B). The proteins are mostly associated with energy metabolism and protein transport (Fig. 5C). Probe targets that were more than twofold enriched are shown in Supplementary Table S1.

To assess which of the probe targets also interact with THC, competition experiments with probe 1 (1 μM) and Δ^8^-THC (10 μM) or Δ^9^-THC (10 μM) were performed. This resulted in one putative protein target of Δ^9^-THC (Cox4i1) and three for Δ^8^-THC (Reep5, Mtch2, Gnb1) (Fig. 5A, D [red dots]) for which the labeling of the protein by probe 1 was reduced by 40–70% (Fig. 5E, Supplementary Table S2). It should be noted that putative protein target Reep5 was enriched only 1.5-fold by probe 1, but is listed because it had the largest reduction after THC-pretreatment (69%±6%). The absence of complete inhibition by THC may be due to a low affinity to these proteins, because an inhibition between 40% and 70% indicates an IC_50_ in the micromolar range. However, as this was measured in presence of 1 μM probe, the actual pK_i_ of THC for these proteins may be a bit higher.

Cox4i1 is involved in energy metabolism, whereas Reep5, Mtch2, and Gnb1 are associated with protein modification and transport, energy metabolism, apoptosis and DNA maintenance, or signal transduction, respectively (Table 1). Interestingly, these four putative protein targets are associated with various neurological diseases as reported in the KEGG and OMIM database (Table 2).^42,43^

**Table 1.** **THC Protein Targets as Identified by Competitive Proteomics**

			Subcellular location					Cellular function					
Gene name	Protein name	% Inhibition±SEM	Nucleus	Mitochondrion	Membrane	Extracellular exosome	Endoplasmic reticulum	Electron transport chain	Post-translational modification/protein folding	Protein transport	Signal transduction	Apoptosis	DNA maintenance
Δ^8^-THC													
Reep5	Receptor expression-enhancing protein 5	69±6			x	x	x		x	x			
Mtch2	Mitochondrial carrier homolog 2	56±7	x	X	x	x		x		x		x	x
Gnb1	Guanine nucleotide-binding protein G_i_,G_s_, G_t_ subunit beta	42±8			x	x					x		
Δ^9^-THC													
Cox4i1	Cytochrome c oxidase subunit 4 isoform 1	46±9	x	X	x	x		x					

Data from this table are shown in Figure 3. Inhibition data are the mean±SEM (*N*=3, *n*=3). Gene ontology data are derived from the Uniprot database, combined with the DAVID Bioinformatics Database.

SEM, standard error of the mean; THC, Δ^9^-Tetrahydrocannabinol.

**Table 2.** **Putative THC Protein Targets with Hits in the KEGG and/or OMIM Database**

Gene name	KEGG pathway	OMIM database		
Δ^8^-THC				
Reep5		Neuropathy, spastic paraplegia		
Gnb1	Ras signaling pathway, Chemokine signaling pathway, PI3K-Akt signaling pathway, Circadian entrainment, Retrograde endocannabinoid signaling, Glutamatergic synapse, Cholinergic synapse, Serotonergic synapse, GABAergic synapse, Dopaminergic synapse, Phototransduction, Morphine addiction, Alcoholism, Pathways in cancer		Mental retardation	Acute somatic leukemia
Δ^9^-THC				
Cox4i1	Oxidative phosphorylation, Metabolic pathways, Cardiac muscle contraction, Nonalcoholic fatty liver disease, Alzheimer's disease, Parkinson's disease, Huntington's disease			

Inhibition data are the mean±SEM (*N*=3, *n*=3). Putative protein targets were analyzed using the KEGG and OMIM database and were enriched ∼2×or more after UV-irradiation.

OMIM, Online Mendelian Inheritance in Man.

## Conclusions

The aim of this study was to identify unknown protein targets of THC using photoaffinity labeling and chemical proteomics. To this end, Δ^8^-THC-derived probe 1 was synthesized in 14 steps with a total yield of 18%. Probe 1 had nanomolar affinity for both CBRs, but was not able to undergo a covalent addition with the CBRs and therefore unable to visualize the CBRs in an established gel-based photoaffinity labeling assay. Different positioning of the photoreactive group in the probe, for example, on the more rigid tricyclic core of the scaffold to enable a stronger interaction between diazirine and amino acid residues, might allow the covalent capturing of CBRs.

Photoaffinity labeling of the proteome of live Neuro2A cells resulted in the identification of ∼150 target proteins. Competition studies with THC significantly reduced enrichment of four proteins by probe 1, which suggests that THC has a limited interaction profile in Neuro2A cells. Reep5, Mtch2, and Gnb1 were identified as putative protein targets of Δ^8^-THC, whereas Cox4i1 was targeted by Δ^9^-THC. These targets are mostly involved in protein handling, energy metabolism, apoptosis or DNA maintenance, which may suggest that long-term exposure of THC may affect a variety of (epigenetic) functions of brain cells. Of note, the affinity and functional activity of THC on these four proteins need to be further validated in orthogonal experiments using recombinant expression systems, followed by experiments to identify a mechanistic link between these proteins and physiological effects of THC.

Taken together, the identification of the putative protein hits described is a first step toward a better understanding of the protein interaction profile of THC, which could ultimately lead to the development of novel therapeutics based on THC.

## Supplementary Material

Supplemental data

Supplemental data

## Abbreviations Used

CBRs: cannabinoid receptors
DMF: dimethylformamide
DMSO: dimethylsulfoxide
FA: formic acid
GPCR: G protein-coupled receptor
HRMS: high resolution mass spectrometry
LC/MS: liquid chromatography-mass spectrometry
MS: multiple sclerosis
NMR: nuclear magnetic resonance
pA*f*BPP: photoaffinity-based protein profiling
PBS: phosphate buffered saline
PEI: polyethyleneimine
rt: room temperature
SDS: sodium dodecyl sulfate
SEM: Standard error of the mean
THC: Δ^9^-Tetrahydrocannabinol
THF: tetrahydrofuran
TLC: thin layer chromatography
UV: ultraviolet
WT: wild type
