# Supplementary material for: Development of a Cannabinoid-Based Photoaffinity Probe to Determine the Δ8/9-Tetrahydrocannabinol Protein Interaction Landscape in Neuroblastoma Cells
Source: Cannabis Cannabinoid Res. 2018 Jul 1;3(1):136–51. doi: 10.1089/can.2018.0003 (PMC6038054; doi:10.1089/can.2018.0003)
Supplement: Supplemental data [file Supp_Table1.pdf]

Supplementary Data

Supplementary Table S1. Putative Protein Targets of Probe 1

Gene name	Protein name	UV-enrichment
Tmem97	Transmembrane protein 97	59.6
Lrrc59	Leucine-rich repeat containing 59	20.3
Slc25a11	Solute carrier family 25 (mitochondrial carrier oxoglutarate carrier), member 11	17.0
Tomm22	Translocase of outer mitochondrial membrane 22 homolog	16.5
Sel1l	Sel-1 suppressor of lin-12-like	11.1
Lbr	lamin B receptor	10.3
Rab15	RAB15, member RAS oncogene family	10.0
Ndufa9	NADH dehydrogenase (ubiquinone) 1 alpha subcomplex, 9	6.5
Mtco2	Cytochrome c oxidase subunit 2	6.5
Slc25a19	Solute carrier family 25 (mitochondrial thiamine pyrophosphate carrier), member 19	5.5
Txndc15	Thioredoxin domain containing 15	5.5
Mtch2	Mitochondrial carrier 2	5.3
Lman2	Lectin, mannose-binding 2	5.3
Vdac2	Voltage-dependent anion channel 2	5.3
Sec11c	SEC11 homolog C, signal peptidase complex subunit	5.1
Rpn2	ribophorin II	5.1
Atp5 j2	ATP synthase, H+ transporting, mitochondrial F0 complex, subunit F2	5.0
Fads2	Fatty acid desaturase 2	5.0
Sgpl1	Sphingosine phosphate lyase 1	4.8
Ssrp1	Structure-specific recognition protein 1	4.7
Rab14	RAB14, member RAS oncogene family	4.5
Atp5o	ATP synthase, H+ transporting, mitochondrial F1 complex, O subunit	4.4
Slc25a39	Solute carrier family 25, member 39)	4.4
Tmem87a	Transmembrane protein 87A	4.3
Gtpbp8	GTP-binding protein 8 (putative)	4.3
Gosr2	Golgi SNAP receptor complex member 2	4.2
Hk1	Hexokinase 1	4.2
Hba	Hemoglobin alpha-chain complex	4.1
Hk2	Hexokinase 2	4.0
Ptges2	Prostaglandin E synthase 2	4.0
Coq3	Coenzyme Q3 methyltransferase	3.9
Steap3	STEAP family member 3	3.9
Atp6v0a1	ATPase, H+ transporting, lysosomal V0 subunit A1	3.7
Mthfd1l	Methylenetetrahydrofolate dehydrogenase (NADP+ dependent) 1-like	3.7
Bnip1	BCL2/adenovirus E1B interacting protein 1	3.7
Tubb5	Tubulin, beta 5 class I	3.5
Cnih4	Cornichon family AMPA receptor auxiliary protein 4	3.4
Usmg5	Upregulated during skeletal muscle growth 5	3.4
Por	P450 (cytochrome) oxidoreductase	3.4
Tubb3	Tubulin, beta 3 class III	3.4
Rab1b	RAB1B, member RAS oncogene family	3.3
Pigs	Phosphatidylinositol glycan anchor biosynthesis, class S	3.3
Yif1b	Yip1 interacting factor homolog B	3.3
Acsl6	Acyl-CoA synthetase long-chain family member 6	3.3
Msmo1	Methylsterol monoxygenase 1	3.2
Ssr3	Signal sequence receptor, gamma	3.2
Iap	Intestinal-type alkaline phosphatase	3.2
Pcyox1	Prenylcysteine oxidase 1	3.1
Mmgt1	Membrane magnesium transporter 1	3.0
Gaa	Glucosidase, alpha, acid	3.0
Uqcrq	Ubiquinol-cytochrome c reductase, complex III subunit VII	3.0
Nup93	Nucleoporin 93	2.9
Epdr1	Ependymin-related protein 1	2.9
Eef1b	Elongation factor 1-beta	2.9
Lgals1	Lectin, galactose binding, soluble 1	2.9
Pgap1	Post-GPI attachment to proteins 1	2.9

(*continued*)

Supplementary Table S1. (Continued)

Gene name	Protein name	UV-enrichment
Mff	Mitochondrial fission factor	2.9
Anxa6	Annexin A6	2.8
Gpr107	G protein-coupled receptor 107	2.8
Adpgk	ADP-dependent glucokinase	2.8
Tmx1	Thioredoxin-related transmembrane protein 1	2.8
Hebp1	Heme binding protein 1	2.8
Acsl1	Acyl-CoA synthetase long-chain family member 1	2.8
Cpox	Coproporphyrinogen oxidase	2.7
Dhrs7	Dehydrogenase/reductase (SDR family) member 7	2.7
Ptdss1	Phosphatidylserine synthase 1	2.7
Surf4	Surfeit gene 4	2.7
Timmdc1	Translocase of inner mitochondrial membrane domain containing 1	2.7
Rdh11	Retinol dehydrogenase 11	2.7
Cers4	Ceramide synthase 4	2.7
Fh	Fetal hematoma	2.7
Slc30a5	Solute carrier family 30 (zinc transporter), member 5	2.7
Vapa	Vesicle-associated membrane protein, associated protein A	2.6
Slc3a2	Solute carrier family 3 (activators of dibasic and neutral amino acid transport), member 2	2.6
Emd	Emerin	2.6
Stom	Stomatin	2.5
Clptm1	Cleft lip and palate-associated transmembrane protein 1	2.5
Uqcrc1	Ubiquinol-cytochrome c reductase core protein 1	2.5
Trp53i11	Transformation-related protein 53 inducible protein 11	2.5
Gtpbp4	GTP-binding protein 4	2.5
Cdipt	CDP-diacylglycerol—inositol 3-phosphatidyltransferase (phosphatidylinositol synthase)	2.5
Hsd17b11	Hydroxysteroid (17-beta) dehydrogenase 11	2.5
Aifm2	Apoptosis-inducing factor, mitochondrion-associated 2	2.5
Sec11a	SEC11 homolog A, signal peptidase complex subunit	2.5
Rab4a	RAB4A, member RAS oncogene family	2.4
Syngr3	Synaptogyrin 3	2.4
Arl6ip5	ADP-ribosylation factor-like 6 interacting protein 5	2.4
Atp2a1	ATPase, Ca++ transporting, cardiac muscle, fast twitch 1	2.4
Pgrmc2	Progesterone receptor membrane component 2	2.4
Tmx3	Thioredoxin-related transmembrane protein 3	2.4
Tmx4	Thioredoxin-related transmembrane protein 4	2.4
Krtcap2	Keratinocyte-associated protein 2	2.4
Cox5a	Cytochrome c oxidase subunit Va	2.4
Wdr74	WD repeat domain 74	2.4
Gnpat	Glyceronephosphate O-acyltransferase	2.4
Copz1	Coatomer protein complex, subunit zeta 1	2.3
Tmem19	Transmembrane protein 19	2.3
Atp2b1	ATPase, Ca++ transporting, plasma membrane 1	2.3
Ncstn	Nicastrin	2.3
Rer1	Retention in endoplasmic reticulum sorting receptor 1	2.3
Vamp3	Vesicle-associated membrane protein 3	2.3
Nptn	Neuroplastin	2.3
Gls	Glutaminase	2.3
Rbm14	RNA binding motif protein 14	2.3
Rack1	Receptor for activated C kinase 1	2.3
Stx7	Syntaxin 7	2.2
Abhd16a	Abhydrolase domain containing 16A	2.2
Slc25a25	Solute carrier family 25 (mitochondrial carrier, phosphate carrier), member 25	2.2
Fam114a2	Family with sequence similarity 114, member A2	2.2
Sec22b	SEC22 homolog B, vesicle trafficking protein	2.2
Kdelr2	KDEL (Lys-Asp-Glu-Leu) endoplasmic reticulum protein retention receptor 2	2.2
Nipsnap1	Nipsnap homolog 1	2.2
Itgb1	Integrin beta 1 (fibronectin receptor beta)	2.2
Letmd1	LETM1 domain containing 1	2.2
Gnb1	Guanine nucleotide binding protein (G protein), beta 1	2.2
Lpcat3	Lysophosphatidylcholine acyltransferase 3	2.2
Syt1	Synaptotagmin I	2.2
Slc25a32	Solute carrier family 25, member 32	2.2
Rab39b	RAB39B, member RAS oncogene family	2.2
Preb	Prolactin regulatory element binding	2.2
Slc16a3	Solute carrier family 16 (monocarboxylic acid transporters), member 3	2.1
Abhd6	Abhydrolase domain containing 6	2.1

(*continued*)

Supplementary Table S1. (Continued)

Gene name	Protein name	UV-enrichment
Rab18	RAB18, member RAS oncogene family	2.1
Hadha	hydroxyacyl-Coenzyme A dehydrogenase/3-ketoacyl-Coenzyme A thiolase/enoyl-Coenzyme A hydratase (trifunctional protein), alpha subunit	2.1
Fam134c	Family with sequence similarity 134, member C	2.1
Slc25a20	Solute carrier family 25 (mitochondrial carnitine/acylcarnitine translocase), member 20	2.1
Uchl1	Ubiquitin carboxy-terminal hydrolase L1	2.1
Hsd17b12	Hydroxysteroid (17-beta) dehydrogenase 12	2.1
Vkorc1l1	Vitamin K epoxide reductase complex, subunit 1-like 1	2.1
Pdhb	Pyruvate dehydrogenase (lipoamide) beta	2.1
Timm17a	Translocase of inner mitochondrial membrane 17a	2.1
Mtnd1	NADH-ubiquinone oxidoreductase chain 1	2.0
Bcap29	B cell receptor-associated protein 29	2.0
Pam16	Presequence translocase-associated motor 16 homolog	2.0
Fkbp8	FK506-binding protein 8	2.0
Cox4i1	Cytochrome c oxidase subunit IV isoform 1	2.0
Reep5	Receptor accessory protein 5	1.5

Proteins that were $>2\times$ abundant after UV-irradiation of probe 1 are shown here. Gene and protein names and the ratio probe versus probe (No UV) of these proteins are listed.

UV, ultraviolet.
