# Supplementary material for: Development of a Cannabinoid-Based Photoaffinity Probe to Determine the Δ8/9-Tetrahydrocannabinol Protein Interaction Landscape in Neuroblastoma Cells
Source: Cannabis Cannabinoid Res. 2018 Jul 1;3(1):136–51. doi: 10.1089/can.2018.0003 (PMC6038054; doi:10.1089/can.2018.0003)
Supplement: Supplemental data [file Supp_Table2.pdf]

Supplementary Table S2. THC Putative Protein Targets with Less Than 50% Inhibition

	Gene name	Protein name	% Inhibition ± SEM
Δ^8-THC	Lin28a	Protein lin-28 homolog A	25 ± 2
	Ssrp1	FACT complex subunit SSRP1	25 ± 7
	Asna1	ATPase Asna1	21 ± 2
	Pdia4	Protein disulfide-isomerase A4	11 ± 3
Δ^9-THC	Rhoc	Rho-related GTP-binding protein RhoC	41 ± 7
	Ldah	Lipid-droplet-associated hydrolase	39 ± 6
	Ppa1	Inorganic pyrophosphatase	39 ± 7

Proteins that were inhibited <50% were excluded from gene ontology analysis. Gene and protein names of these proteins are listed here. SEM, standard error of the mean.
